# Cochlear Implant Fitting Profiles in Children with Congenital Cytomegalovirus: One-Year Longitudinal Comparison with *GJB2*-Related Hearing Loss

**DOI:** 10.3390/children13101283

**Published:** 2026-09-22

**Authors:** Sara Ghiselli, Patrizia Consolino, Diego Di Lisi, Patrizia Frontera, Alessia Ghia, Domenico Cuda

**Affiliations:** 1 Department of Medicine and Surgery, University of Parma, 43126 Parma, Italy or d.cuda@ausl.pc.it; 2 Audiology Department, AUSL Piacenza, 29121 Piacenza, Italy; p.frontera@ausl.pc.it; 3 SSD Audiologia e Impianti Cocleari, ASL Città di Torino, 10141 Torino, Italy; patrizia.consolino@aslcittaditorino.it (P.C.); diego.dilisi@aslcittaditorino.it (D.D.L.); 4 SS. Antonio e Biagio e Cesare Arrigo Civil Hospital, 15121 Alessandria, Italy; alessia.ghia@aou.al.it

**Keywords:** congenital cytomegalovirus, cCMV, cochlear implant, CI fitting, eCAP levels, electrode impedance, *GJB2*, audiological outcomes

## Abstract

**Highlights:**

**What are the main findings?**

- Longitudinal mixed-effects models identified time-dependent differences in eCAP, electrode impedance, and behavioural fitting measures among children with cCMV-related and *GJB2*-related hearing loss.
- Aided PTA and CAP scores improved over time; children with cCMV and MRI abnormalities had lower CAP scores than both the *GJB2* and cCMV groups without MRI abnormalities.

**What are the implications of the main findings?**

- Cochlear implant fitting profiles vary according to hearing-loss aetiology; therefore, eCAP thresholds should be interpreted within each child’s behavioural and developmental context.
- Objective fitting measures, aided audiometry, and functional auditory assessments should be integrated when programming and monitoring children with cCMV-related hearing loss.

**Abstract:**

**Background/Objectives**: Congenital cytomegalovirus (cCMV) infection is a major cause of paediatric sensorineural hearing loss and may involve peripheral and central auditory pathways. This study investigated longitudinal cochlear implant (CI) fitting parameters in children with cCMV-related hearing loss, including the influence of MRI-detectable brain abnormalities, using *GJB2*-related hearing loss as a reference. **Methods**: This retrospective multicentre study included 56 children (89 implanted ears) divided into cCMV, altered cCMV (with MRI abnormalities), and *GJB2* groups. Outcomes were evaluated at activation and 1, 3, 6, and 12 months after activation using longitudinal mixed-effects models with participant and ear nested within participant as random intercepts. **Results**: Significant group-by-time interactions were identified for eCAP, impedance, C and T levels, and dynamic range. Adjusted eCAP differences were time-specific rather than uniform throughout follow-up. C and T levels showed no significant adjusted between-group contrasts, and dynamic range differed only between cCMV and *GJB2* at 1 month. Aided PTA improved over time and was better in the cCMV group than in the other groups. CAP scores improved over time and were lower in the altered cCMV group than in both the *GJB2* and cCMV groups. **Conclusions**: CI fitting parameters followed different longitudinal patterns across aetiological groups, but most between-group differences were time-specific. MRI abnormalities were associated with poorer functional auditory performance rather than a uniform electrophysiological profile. Objective fitting measures, aided audibility, and functional auditory performance should therefore be interpreted jointly during follow-up.

## 1. Introduction

Congenital cytomegalovirus (cCMV) is the most common congenital infection in industrialised countries, with an estimated global birth prevalence of 0.64% [1]. Although many infected newborns are asymptomatic or paucisymptomatic, permanent sequelae may develop in 17–20% of cases [2].

Central nervous system involvement is one of the most relevant manifestations of cCMV. Brain imaging may reveal ventriculomegaly, periventricular cysts, cerebral atrophy or hypoplasia, intracranial calcifications, and other structural abnormalities [3,4]. These findings are often associated with neurological signs and cognitive or motor impairment [5].

Hearing loss is another major sequela of cCMV and has a substantial impact on communication, language development, and quality of life. It occurs in approximately 50% of symptomatic cases and 10% of asymptomatic cases, making cCMV the leading non-genetic cause of paediatric sensorineural hearing loss [6]. cCMV-related hearing loss is clinically heterogeneous: it may be unilateral or bilateral, range from mild to profound, present with early or delayed onset, and follow a fluctuating or progressive course. Because of this variability and the risk of progression, children with cCMV require regular audiological monitoring from birth to school age, with assessments at least every six months. Early detection is essential to enable timely rehabilitation with hearing aids or, in severe-to-profound cases, cochlear implantation.

Cochlear implantation (CI) is an established treatment for severe-to-profound sensorineural hearing loss. After implantation, device performance depends on repeated programming, or fitting, which aims to optimise electrical stimulation according to objective and behavioural fitting measures.

Neural response telemetry, based on electrically evoked compound action potentials (eCAPs), is one of the most widely used objective fitting measures. eCAPs reflect the electrically evoked response of auditory nerve fibres recorded through the intracochlear electrodes. Behavioural fitting measures include threshold (T) and comfort (C) levels. The T level represents the minimum current required on each electrode to elicit an auditory sensation, whereas the C level represents the maximum current that can be delivered while maintaining comfortable listening. Together, eCAP thresholds, T levels, C levels, and the resulting dynamic range provide complementary information for programming each electrode of the cochlear implant array.

Although both objective and behavioural measures can usually be obtained in adults, paediatric CI fitting often relies heavily on objective measures because young children may have limited attention and cooperation. eCAPs are particularly useful in this setting, as their thresholds generally fall within the behavioural dynamic range, at approximately 67% of the range between T and C levels [7]. CI fitting parameters may provide clinically relevant information about the cochlear electrode–neural interface, but they may also be influenced by central auditory pathway involvement, as suggested in children with autism spectrum disorder, brain abnormalities, or psychomotor developmental delay [8,9].

This distinction is particularly relevant in children with cCMV, as the underlying auditory dysfunction may involve not only cochlear structures but also the auditory nerve and central auditory pathways. By contrast, *GJB2*-related hearing loss is generally considered a predominantly peripheral cochlear disorder. Pathogenic variants in *GJB2*, which encodes the gap-junction protein connexin 26, are among the most common genetic causes of autosomal recessive non-syndromic sensorineural hearing loss [10]. The resulting hearing loss is typically bilateral and congenital or prelingual, although its severity and clinical course may vary according to genotype [11]. Because cochlear function is primarily affected, with relative preservation of the auditory nerve and central auditory pathways, children with severe-to-profound *GJB2*-related hearing loss generally achieve favourable auditory and speech-perception outcomes after cochlear implantation, although individual variability remains [12,13]. These characteristics make *GJB2*-related hearing loss a clinically meaningful comparison condition for investigating features that may be specifically associated with cCMV.

Nevertheless, it remains unclear whether longitudinal cochlear implant fitting patterns in children with cCMV differ from those observed in children with *GJB2*-related hearing loss. Addressing this question is clinically relevant because CI fitting parameters may provide indirect insight into whether cCMV-related auditory dysfunction exhibits electrophysiological characteristics that differ from those of a predominantly peripheral cochlear disorder. However, longitudinal data on objective and behavioural fitting profiles in children with cCMV remain scarce, comparisons with an appropriate cochlear reference group are limited, and the association between MRI-detectable brain abnormalities and fitting parameters remains poorly understood.

Therefore, this study aimed to analyse longitudinal changes in objective and behavioural CI fitting parameters in children with cCMV-related hearing loss, to compare these profiles with those observed in children with *GJB2*-related hearing loss as a cochlear reference group, and to assess whether MRI-detectable brain abnormalities are associated with distinct fitting profiles or audiological outcomes.

## 2. Materials and Methods

### 2.1. Study Design

This was a retrospective observational multicentre study. Written informed consent was obtained from parents or legal guardians. The study was approved by the local institutional ethics committee Comitato Etico dell’Area Vasta Emilia Nord (AVEN) (N. 387/2024/OSS/AUSLPC on 25 September 2024).

### 2.2. Sampling Criteria

The subjects were required to meet the following criteria:

- Severe-to-profound hearing loss;
- Hearing loss associated with cCMV infection or *GJB2* mutations;
- Cochlear implantation performed before 18 years of age;
- Preoperative brain MRI available;
- Absence of middle or inner ear malformations;
- First CI surgery;
- Written informed consent provided by parents or legal guardians.

The exclusion criteria were as follows:

- Use of electroacoustic stimulation on the implanted side;
- Cochlear implant revision surgery.

### 2.3. Participants

A total of 56 subjects (<18 years of age) who underwent cochlear implantation for hearing loss secondary to congenital cytomegalovirus (cCMV) infection or *GJB2* gene mutations were included in the study. In subjects with bilateral cochlear implants, each implanted ear with available longitudinal data was analysed, resulting in 89 implanted ears.

The preoperative PTA, calculated at 500, 1000, 2000, and 4000 Hz, was 118.7 dB (SD 11.5; range 95–130 dB) in the implanted ear and 113.4 dB (SD 19.3; range 10–130 dB) in the contralateral ear.

Of the 56 children, 38 underwent bilateral cochlear implantation, 12 used bimodal stimulation, and six underwent unilateral implantation. Both implanted ears were available for analysis in 33 bilaterally implanted children; for five bilaterally implanted children, longitudinal data were available for only one ear. Overall, 89 implanted ears were included.

All patients received Cochlear™ (Sydney, Australia) cochlear implants with perimodiolar electrode arrays (29 CI24RE [CA], 17 CI512, 13 CI532, 8 CI612, and 22 CI632) and behind-the-ear sound processors.

The mean age at cochlear implantation was 2.8 years (SD 2.7; range 1–12 years).

All subjects underwent preoperative brain magnetic resonance imaging (MRI). MRI findings were considered abnormal when one or more of the following abnormalities were detected: parenchymal lesions, white matter myelination abnormalities, intracranial calcifications, polymicrogyria, microcephaly, cerebral hypoplasia, hemispheric asymmetry, or cerebral atrophy.

The study population was divided into three groups according to aetiology and the presence of MRI-detectable brain abnormalities:

1. cCMV group: subjects with confirmed congenital CMV infection and no MRI-detectable brain abnormalities.
2. Altered cCMV group: subjects with confirmed congenital CMV infection and MRI-detectable brain abnormalities.
3. *GJB2* group: subjects with confirmed *GJB2*-related hearing loss and no MRI-detectable brain abnormalities.

Among the 27 children with cCMV, the median age at first cochlear implantation was 1.0 year (IQR, 1.0–2.25; range, 1–7 years). The median age was 1.0 year (IQR, 1.0–1.75; range, 1–7 years) in children without MRI abnormalities and 1.25 years (IQR, 1.0–2.63; range, 1–7 years) in those with MRI abnormalities. In the *GJB2* group, the median age at first cochlear implantation was 1.0 year (IQR, 1.0–2.0; range, 1–8 years).

The characteristics of the cCMV group, altered cCMV group, and *GJB2* group are summarised in Table 1.

### 2.4. CI Fitting and Audiological Outcome Assessment

All subjects were evaluated at CI activation (T0) and at the scheduled follow-up visits 1 (T1), 3 (T3), 6 (T6), and 12 (T12) months after activation. At each visit, all subjects underwent CI fitting and audiological outcome assessments agreed upon by the two centers.

CI fitting included objective measures (electrically evoked compound action potentials [eCAPs] and electrode impedance) and behavioural fitting measures (threshold [T] levels, comfort [C] levels, and dynamic range) for each electrode of the CI array. For eCAP levels, electrode impedance, and T and C levels, the mean value across all electrodes was calculated at each follow-up visit. Fitting procedures were standardized across the two participating centres and were performed using the manufacturer’s software (Custom Sound Pro, version 7.0) with the following parameters: Advanced Combination Encoder (ACE) coding strategy, 10 maxima, and a pulse width of 25 μs.

Outcome measures included the Categories of Auditory Performance (CAP) score and aided free-field pure-tone average (PTA) with the cochlear implant. PTA was considered as the average air-tonal threshold at 500, 1000, 2000, and 4000 Hz frequencies and was performed in aided free field conditions.

In subjects with asymmetric hearing loss or single-sided deafness (SSD), masking of the contralateral ear was applied during audiometric testing.

The CAP score is derived from a hierarchical ordinal scale used to assess functional auditory performance in everyday life. It evaluates the ability to detect, discriminate, and understand sounds in increasingly complex listening situations, ranging from basic awareness of environmental sounds to the ability to understand conversational speech without visual cues. The scale comprises eight categories (0–7), with lower scores indicating minimal auditory perception and higher scores reflecting more advanced auditory abilities, including the understanding of speech in both quiet and noisy environments without lip-reading. CAP scores were obtained through structured interviews with parents or legal guardians conducted by qualified professionals, including audiologists, otorhinolaryngologists, and speech-language therapists, at the two participating centres.

Although both centres followed their routine clinical procedures, no additional study-specific standardization of outcome assessment was implemented.

### 2.5. Statistical Analysis

Continuous longitudinal outcomes (eCAP levels, electrode impedance, T levels, C levels, dynamic range, and aided PTA) were analysed using linear mixed-effects models. Group (cCMV, altered cCMV, and *GJB2*), follow-up time (T0, T1, T3, T6, and T12), and the group-by-time interaction were included as fixed effects. Participant and implanted ear nested within participant were included as random intercepts to account for the correlation between ears from the same child and repeated observations from the same ear. Follow-up time was treated as a categorical variable. Models were fitted by maximum likelihood, and omnibus effects were assessed using likelihood-ratio tests.

When indicated by the omnibus tests, estimated marginal means were used for pairwise comparisons between groups at each follow-up time and between follow-up times within each group. Effect estimates are reported as mean differences (MDs) with 95% confidence intervals (CIs). *p* values were adjusted for multiple testing using the Holm method. Model assumptions were assessed by inspection of residual-versus-fitted and normal Q-Q plots. As a sensitivity analysis, all models were repeated using one ear per participant, selecting a priori as the ear with the lowest numeric study ID, to verify that inclusion of both ears did not inflate statistical significance.

Aided PTA was analysed as a continuous outcome using a longitudinal linear mixed-effects model. Although CAP is an ordinal scale, its eight ordered categories (0–7) were treated as approximately continuous and analysed using the same mixed-effects model. For both outcomes, group, categorical follow-up time, and the group-by-time interaction were included as fixed effects, while participant and implanted ear nested within participant were included as random intercepts. This modelling assumption for CAP was considered when interpreting the results and is acknowledged as a study limitation. Statistical significance was set at *p* < 0.05.

Analyses were conducted in Python 3.12 using NumPy 2.3.5, SciPy 1.17.0, and pandas 2.2.3 [14,15].

## 3. Results

The longitudinal mixed-effects model for eCAP levels identified a significant group-by-time interaction (likelihood-ratio test: χ^2^(8) = 31.36, *p* < 0.001), indicating that between-group differences varied across follow-up.

Holm-adjusted comparisons showed higher eCAP levels in the cCMV group than in the *GJB2* group at T0 (MD = 16.19, 95% CI 8.45 to 23.94, *p* < 0.001), T6 (MD = 14.36, 95% CI 6.62 to 22.11, *p* < 0.001), and T12 (MD = 10.79, 95% CI 2.98 to 18.60, *p* = 0.020). The altered cCMV group had higher eCAP levels than the *GJB2* group at T0 (MD = 8.01, 95% CI 1.30 to 14.73, *p* = 0.039), while the cCMV group had higher levels than the altered cCMV group at T6 (MD = 11.27, 95% CI 2.67 to 19.86, *p* = 0.020). No other adjusted between-group contrasts were significant. Relative to T0, eCAP levels decreased at T1, T3, and T12 in the cCMV group and at all follow-up visits in the altered cCMV group; no significant temporal contrasts were found in the *GJB2* group (Figure 1).

The mixed-effects model for mean electrode impedance identified a significant group-by-time interaction (χ^2^(8) = 17.31, *p* = 0.027), demonstrating that longitudinal changes in impedance differed among the three groups.

At T0, impedance was lower in the altered cCMV group than in the *GJB2* group (MD = −1.92 kΩ, 95% CI −2.76 to −1.08, *p* < 0.001); no other adjusted between-group contrasts were significant. In all three groups, impedance was significantly lower at every follow-up visit than at T0 (all adjusted *p* < 0.001) (Figure 2).

Longitudinal mixed-effects models identified significant group-by-time interactions for C levels (χ^2^(8) = 47.14, *p* < 0.001), T levels (χ^2^(8) = 33.17, *p* < 0.001), and dynamic range (χ^2^(8) = 24.78, *p* = 0.002). These interactions indicate that the trajectories of the behavioural fitting measures differed among groups.

No adjusted between-group contrasts were significant for C or T levels. For dynamic range, the cCMV group had a higher value than the *GJB2* group only at T1 (MD = 5.96 CL, 95% CI 2.01 to 9.91, *p* = 0.009); the remaining adjusted between-group contrasts were not significant.

C levels and dynamic range increased from T0 at every follow-up visit in all groups (all adjusted *p* < 0.001). T levels increased at every follow-up visit in the *GJB2* and cCMV groups (all adjusted *p* < 0.001); in the altered cCMV group, increases were significant at T3 (*p* = 0.047), T6 (*p* = 0.047), and T12 (*p* = 0.031), but not at T1 (Figure 3).

For aided PTA, the group-by-time interaction was not significant (χ^2^(8) = 13.31, *p* = 0.102). In the additive model, significant effects were observed for group (χ^2^(2) = 11.95, *p* = 0.003) and time (χ^2^(4) = 320.94, *p* < 0.001), indicating progressive improvement over follow-up with overall differences among groups.

Aided PTA was lower (better) in the cCMV group than in the *GJB2* group (MD = −7.93 dB, 95% CI −12.32 to −3.54, adjusted *p* = 0.001) and the altered cCMV group (MD = −7.48 dB, 95% CI −12.38 to −2.58, adjusted *p* = 0.006); the altered cCMV and *GJB2* groups did not differ significantly. Relative to T0, aided PTA improved at every follow-up visit (all adjusted *p* < 0.001) (Figure 4).

For CAP scores, the group-by-time interaction was not significant (χ^2^(8) = 6.68, *p* = 0.571). The additive model showed significant effects of group (χ^2^(2) = 10.00, *p* = 0.007) and time (χ^2^(4) = 499.23, *p* < 0.001). The altered cCMV group had lower CAP scores than the *GJB2* group (MD = −1.00, 95% CI −1.61 to −0.39, adjusted *p* = 0.004) and the cCMV group (MD = −0.92, 95% CI −1.70 to −0.15, adjusted *p* = 0.039); the cCMV and *GJB2* groups did not differ significantly. CAP scores increased from T0 at every follow-up visit (all adjusted *p* < 0.001) (Figure 5).

### Sensitivity Analysis

Repeating the models with one prespecified ear per participant (56 participants and 56 ears) confirmed the principal findings for aided PTA, CAP, eCAP, C levels, T levels, and dynamic range. The group-by-time interactions remained significant for eCAP (*p* = 0.002), C levels (*p* < 0.001), T levels (*p* = 0.004), and dynamic range (*p* = 0.015); the interactions remained non-significant for PTA (*p* = 0.162) and CAP (*p* = 0.614). For impedance, however, the group-by-time interaction was attenuated and no longer statistically significant (*p* = 0.082), and the additive group effect was also non-significant (*p* = 0.144), whereas the effect of time remained significant (*p* < 0.001) (Table 2).

## 4. Discussion

This study provides evidence that cochlear implant fitting profiles evolve differently over time according to hearing-loss aetiology. Mixed-effects modelling showed significant group-by-time interactions for eCAP, impedance, C and T levels, and dynamic range. However, multiplicity-adjusted comparisons indicated that most between-group differences were confined to specific follow-up visits rather than being uniformly present throughout the first year.

For eCAP, the cCMV group showed higher levels than the *GJB2* group at activation, 6 months, and 12 months, whereas the altered cCMV group differed from *GJB2* only at activation. A difference between the two cCMV groups was observed only at 6 months. These time-specific findings may reflect differences in the electrode-neural interface and/or neural excitability, but they do not support a uniform cCMV-specific elevation across every follow-up visit.

The limited and time-dependent differences between the two cCMV groups suggest that MRI-detectable abnormalities do not define a stable eCAP phenotype. Conventional MRI provides a macroscopic assessment and may not detect subtle cochlear or auditory-pathway involvement. Because cCMV may affect multiple levels of the auditory system [16], the observed eCAP trajectories should be interpreted as exploratory evidence of heterogeneous neural and electrode-neural interface responses rather than as a direct marker of central abnormalities.

Impedance decreased significantly from activation in all groups. The only adjusted between-group difference in the primary analysis occurred at activation, when the altered cCMV group had lower impedance than the *GJB2* group. This finding was attenuated in the one-ear sensitivity analysis; consequently, the evidence for an aetiology-related impedance difference should be interpreted cautiously.

These results are broadly consistent with Zajdel et al. [17], who reported no sustained differences in mean electrode impedance between cCMV and non-cCMV ears during follow-up. Both studies provide no evidence of persistently increased impedance in cCMV-related hearing loss.

In addition, all groups showed a marked decrease in electrode impedance from cochlear implant activation (T0) to the first month of use (T1), after which electrode impedance remained stable throughout the follow-up period. This temporal pattern is consistent with previous studies showing that electrode impedance typically increases during the early postoperative period, decreases following implant activation and electrical stimulation, and subsequently stabilises over the following weeks or months [18,19]. A similar longitudinal pattern has also been reported in children with cCMV, with an initial increase in electrode impedance around activation followed by a decrease and subsequent stabilization during CI use [17]. Taken together, these findings provide no evidence of an abnormal or sustained increase in electrode impedance in cCMV-related hearing loss.

Behavioural fitting measures increased over time, but adjusted between-group contrasts were sparse: C and T levels did not differ significantly between groups, and dynamic range differed only between cCMV and *GJB2* at 1 month. Reliable behavioural measures may be difficult to obtain in young children; objective measures such as eCAP therefore remain useful for programming, but neither objective nor behavioural values should be interpreted as stable aetiology-specific signatures.

Aided PTA and CAP scores improved over time without significant group-by-time interactions. The cCMV group had better overall aided PTA than both the altered cCMV and *GJB2* groups. In contrast, the altered cCMV group had lower overall CAP scores than both comparison groups, while cCMV and *GJB2* did not differ significantly in CAP.

These apparently divergent findings highlight the distinction between aided audibility and functional auditory performance. Aided PTA primarily reflects access to and detection of auditory stimuli, whereas CAP scores assess the functional use of auditory information in increasingly complex everyday listening situations. Therefore, better aided PTA does not necessarily translate into equivalent functional auditory performance.

The lower CAP scores in the altered cCMV group, despite the absence of a consistent corresponding electrophysiological pattern, suggest that MRI-detectable abnormalities may be more closely related to functional processing and use of auditory information than to peripheral electrically evoked responses.

Previous studies have consistently shown that cochlear implantation is an effective intervention for providing auditory access in children with cCMV-related hearing loss [20,21]. However, considerable variability in audiological outcomes has been reported, particularly in relation to the presence and severity of associated comorbidities and neurodevelopmental involvement [17,22].

This variability is also reflected in the systematic review by Kraaijenga et al. [23], which found that children with cCMV frequently achieved poorer cochlear implant outcomes than non-cCMV paediatric implant users. Importantly, poorer performance was largely associated with cCMV-related comorbidities, whereas the available evidence did not indicate consistently unfavourable outcomes in children with asymptomatic cCMV. This distinction is consistent with our observation that CAP scores were lowest in the altered cCMV group, comprising children with MRI-detectable brain abnormalities, whereas children with cCMV without such abnormalities showed better functional auditory performance. Collectively, these findings suggest that variability in cochlear implant outcomes among children with cCMV may be related not only to the infection itself but also to the extent of associated neurological and neurodevelopmental involvement.

Our study extends the available evidence by showing that variability in cCMV-related cochlear implantation also involves objective and behavioural fitting parameters. Importantly, differences in fitting parameters did not consistently parallel differences in aided PTA or CAP scores, suggesting that electrophysiological fitting measures are complementary to, rather than directly predictive of, functional auditory performance. Objective fitting measures, aided audibility, and functional auditory performance may therefore represent distinct but complementary dimensions of cochlear implant outcomes in children with cCMV.

Clinicians should be aware that objective fitting measures may follow different time-dependent trajectories. eCAP thresholds should therefore be interpreted within each child’s behavioural, longitudinal, and developmental context rather than used in isolation to predict behavioural levels or functional outcomes. Similarly, good aided PTA should not necessarily be interpreted as evidence of equivalent functional auditory development.

The observed differences between the aetiological groups may also have been influenced by clinical characteristics that were not included as covariates in the present models. Age at implantation may affect subsequent auditory development, while baseline hearing status may influence the magnitude and trajectory of improvement after cochlear implantation. Outcomes may also differ according to hearing configuration, including unilateral cochlear implantation, bilateral cochlear implantation, and bimodal stimulation. Given the limited sample size and unequal group distribution, simultaneous adjustment for these variables would have increased the risk of overfitting and unstable estimates. Residual confounding therefore cannot be excluded, and the observed differences should not be attributed exclusively to hearing-loss aetiology. Larger prospective studies are needed to assess the independent contributions of aetiology, age at implantation, baseline hearing, and hearing configuration.

This study has several limitations. Its retrospective design may have introduced selection and information biases, while the relatively small sample size, particularly within the cCMV subgroups, limited the precision and statistical power of time-specific comparisons and precluded stable adjustment for multiple clinical covariates. MRI abnormalities were heterogeneous in type and severity. CAP is an ordinal measure but was treated as approximately continuous in the mixed-effects analysis; this modelling assumption should therefore be considered when interpreting the findings. Although the primary models accounted for the correlation between ears, the one-ear sensitivity analysis had lower precision and indicated that the impedance interaction was not robust. Finally, despite the use of standardized fitting parameters, the multicentre and retrospective design may have introduced residual variability in clinical management, rehabilitation, and outcome assessment procedures.

## 5. Conclusions

In conclusion, CI fitting parameters showed different longitudinal trajectories across cCMV, altered cCMV, and *GJB2* groups, but most adjusted between-group differences were limited to specific follow-up visits. Aided audibility improved in all groups, whereas CAP scores were poorer in children with cCMV and MRI-detectable abnormalities. Objective fitting measures, aided PTA, and functional auditory performance therefore capture complementary dimensions and should be interpreted jointly. Further prospective studies are needed to clarify the relationship between fitting trajectories, neural involvement, and long-term outcomes.

## Figures and Tables

**Figure 1:**
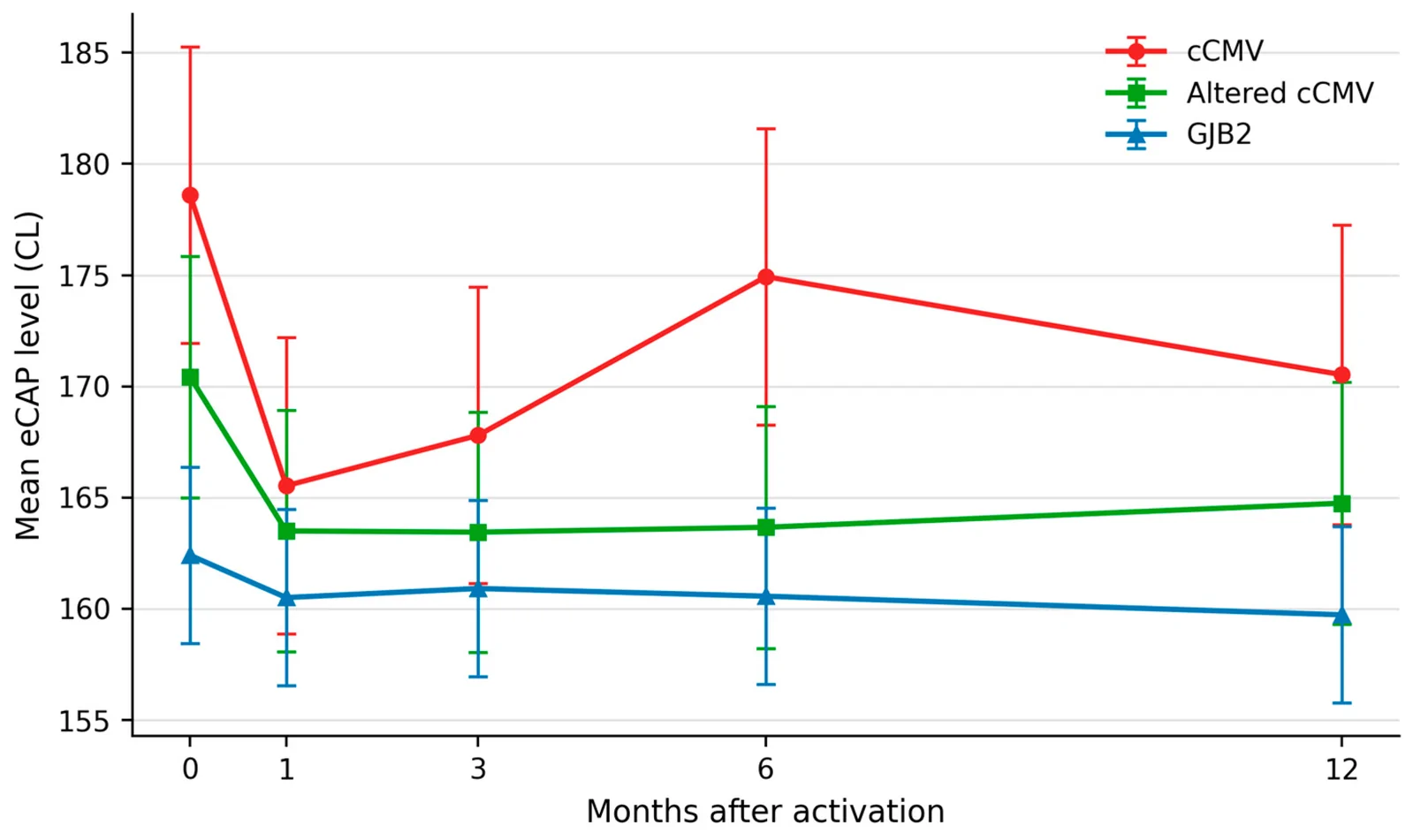
Model-estimated marginal means and 95% confidence intervals for eCAP levels in the cCMV group (red circles), altered cCMV group (green squares), and *GJB2* group (blue triangles) at T0, T1, T3, T6, and T12.

**Figure 2:**
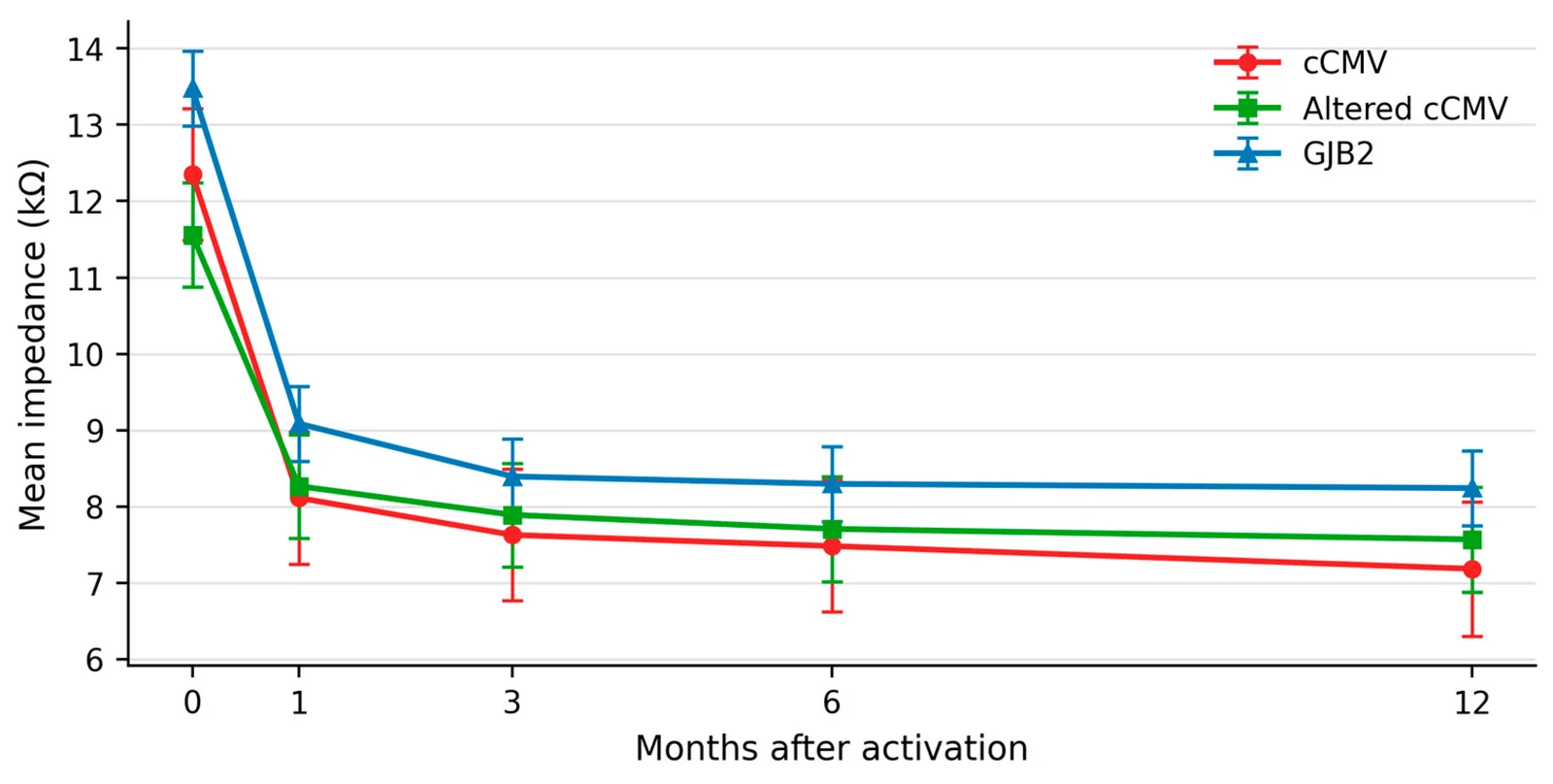
Model-estimated marginal means and 95% confidence intervals for electrode impedance in the cCMV group (red circles), altered cCMV group (green squares), and *GJB2* group (blue triangles) at T0, T1, T3, T6, and T12.

**Figure 3:**
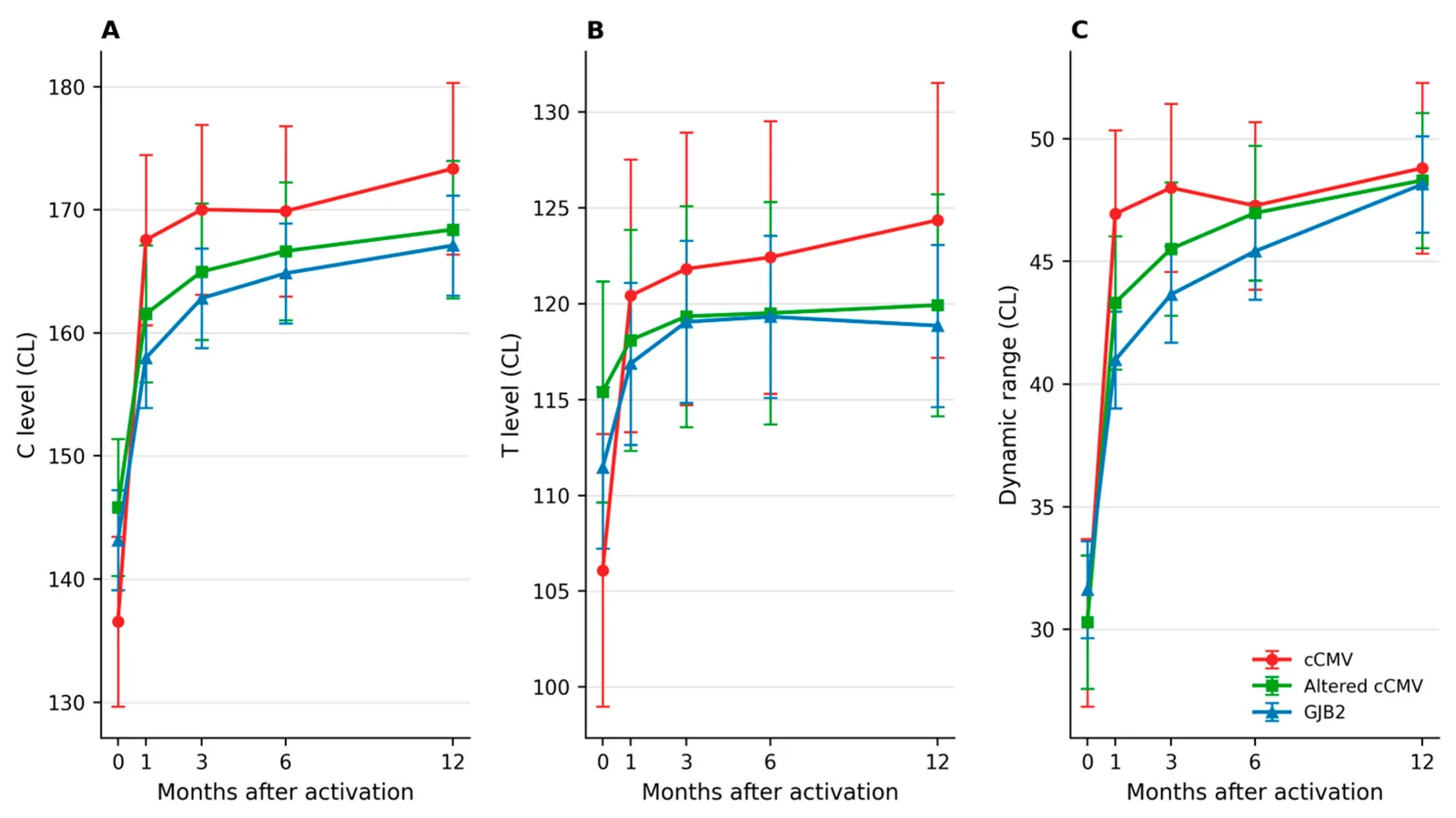
Model-estimated marginal means and 95% confidence intervals for C levels (**A**), T levels (**B**), and dynamic range (**C**) in the cCMV group (red circles), altered cCMV group (green squares), and *GJB2* group (blue triangles) at T0, T1, T3, T6, and T12.

**Figure 4:**
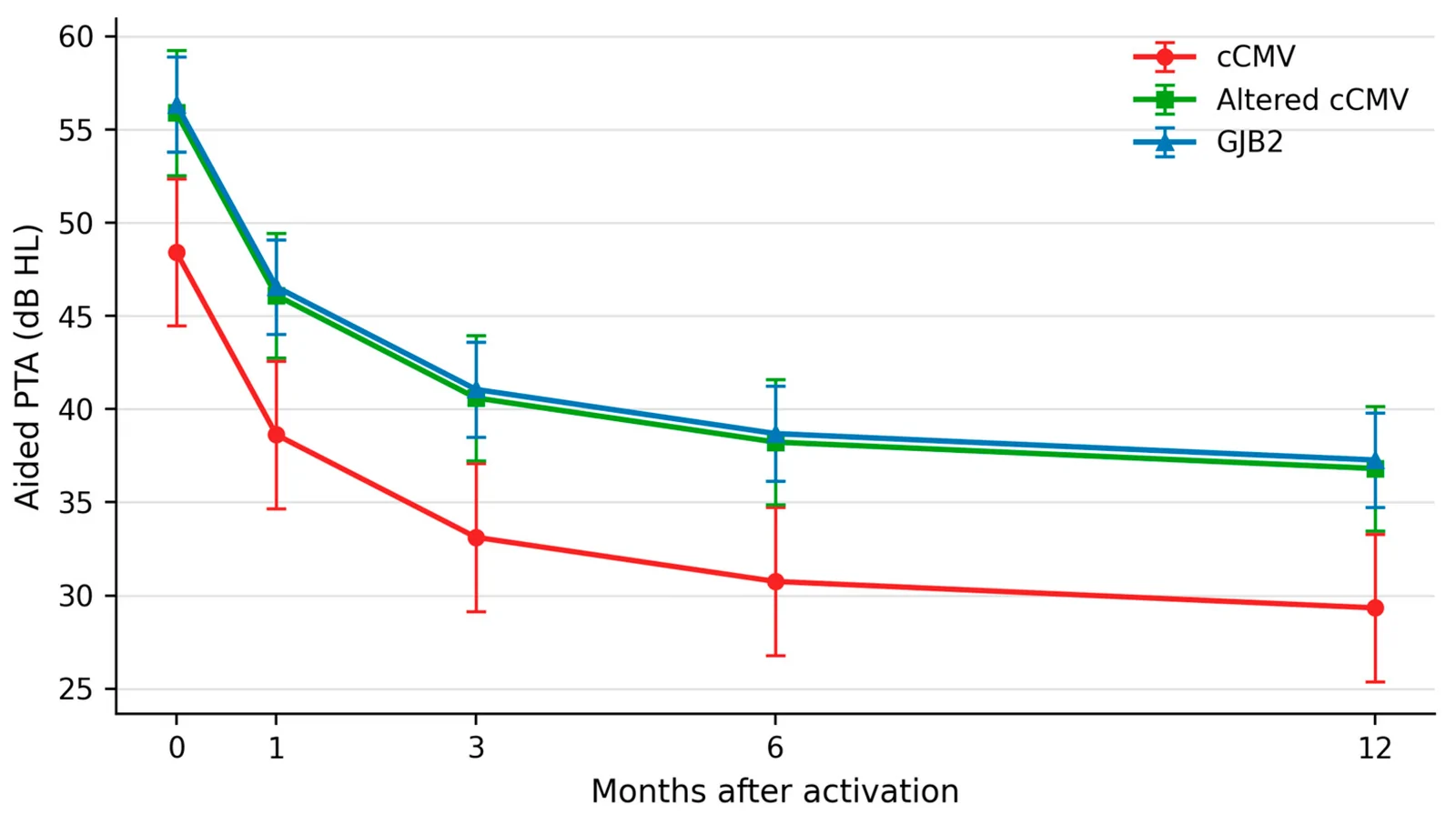
Model-estimated marginal means and 95% confidence intervals for aided PTA in the cCMV group (red circles), altered cCMV group (green squares), and *GJB2* group (blue triangles) at T0, T1, T3, T6, and T12.

**Figure 5:**
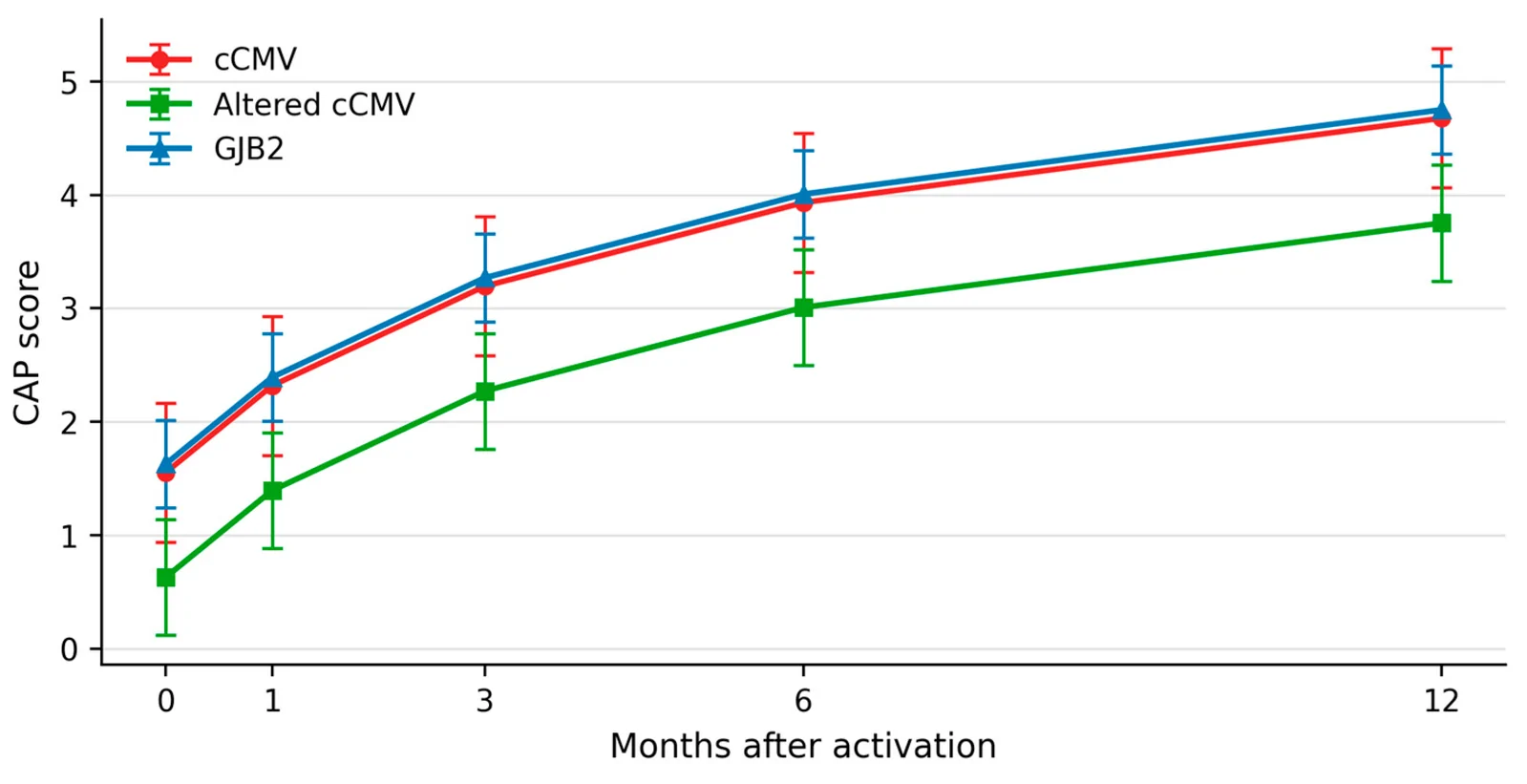
Model-estimated marginal means and 95% confidence intervals for CAP scores in the cCMV group (red circles), altered cCMV group (green squares), and *GJB2* group (blue triangles) at T0, T1, T3, T6, and T12. CAP was treated as approximately continuous in the mixed-effects model.

**Table 1:** Characteristics of the three study groups.

	cCMV Group	Altered cCMV Group	*GJB2* Group
Subjects; implanted ears	11; 15	16; 25	29; 49
Sex (male/female)	5:6	7:9	14:15
Implanted side (right/left)	7:8	14:11	29:20
Stimulation mode (bimodal/bilateral/unilateral), participants	3:7:1 *	3:11:2	6:20:3
Age at first CI surgery, years, median (IQR; range)	1.0 (IQR, 1.0–1.75; range, 1–7 years)	1.25 (IQR, 1.0–2.63; range, 1–7 years)	1.0 (IQR, 1.0–2.0; range, 1–8)
Pre-CI PTA (dB)	124.2 (SD 11.0; range 100–130)	121.4 (SD 11.4; range 100–130)	113.1 (SD 8.94; range 85–130)

* Subject with CI for single-sided deafness (SSD).

**Table 2:** Significant Holm-adjusted between-group contrasts from the primary mixed-effects models. MD, mean difference; CI, confidence interval; CL, clinical current level.

Outcome/Time	Contrast	MD (95% CI)	Adjusted *p*
PTA, overall	cCMV—*GJB2*	−7.93 dB (−12.32 to −3.54)	0.001
PTA, overall	cCMV—altered cCMV	−7.48 dB (−12.38 to −2.58)	0.006
CAP, overall	altered cCMV—*GJB2*	−1.00 (−1.61 to −0.39)	0.004
CAP, overall	altered cCMV—cCMV	−0.92 (−1.70 to −0.15)	0.039
eCAP, T0	cCMV—*GJB2*	16.19 CL (8.45 to 23.94)	<0.001
eCAP, T0	altered cCMV—*GJB2*	8.01 CL (1.30 to 14.73)	0.039
eCAP, T6	cCMV—*GJB2*	14.36 CL (6.62 to 22.11)	<0.001
eCAP, T6	cCMV—altered cCMV	11.27 CL (2.67 to 19.86)	0.020
eCAP, T12	cCMV—*GJB2*	10.79 CL (2.98 to 18.60)	0.020
Dynamic range, T1	cCMV—*GJB2*	5.96 CL (2.01 to 9.91)	0.009
Impedance, T0	altered cCMV—*GJB2*	−1.92 kΩ (−2.76 to −1.08)	<0.001

## Data Availability

The data presented in this study are only available on request from the corresponding author due to privacy restrictions.

## Abbreviations

The following abbreviations are used in this manuscript:

cCMV	Congenital cytomegalovirus
eCAPs	Evoked compound action potentials
CI	Cochlear Implant
